# Health-Related Quality of Life and Associated Factors of Frontline Railway Workers: A Cross-Sectional Survey in the Ankang Area, Shaanxi Province, China

**DOI:** 10.3390/ijerph13121192

**Published:** 2016-11-30

**Authors:** Xiaona Zhang, Gang Chen, Feng Xu, Kaina Zhou, Guihua Zhuang

**Affiliations:** 1 Department of Epidemiology and Biostatistics, School of Public Heath, Xi’an Jiaotong University Health Science Center, No. 76 West Yanta Road, Xi’an 710061, Shaanxi, China; ppflyman1@stu.xjtu.edu.cn; 2 School of Medicine, Flinders University, Adelaide, SA 5042, Australia; gang.chen@flinders.edu.au; 3 Xi’an Railway Center for Disease Control and Prevention, Xi’an 710054, Shaanxi, China; hf8876303@163.com; 4 Department of Nursing, Xi’an Jiaotong University Health Science Center, No. 76 West Yanta Road, Xi’an 710061, Shaanxi, China; dfyq100@163.com

**Keywords:** China, frontline railway worker, quality of life, risk factor, Short Form 36 version 2

## Abstract

After validation of the widely used health-related quality of life (HRQOL) generic measure, the Short Form 36 version 2 (SF-36v2), we investigated the HRQOL and associated factors of frontline railway workers in China. Ground workers, conductors, and train drivers were selected from Ankang Precinct by using a stratified cluster sampling technique. A total of 784 frontline railway workers participated in the survey. The reliability and validity of SF-36v2 was satisfactory. The physical component summary (PCS) scores of three subgroups attained the average range for the USA general population, whereas the mental component summary (MCS) scores were well below the range. Most domains scored below the norm, except for the physical functioning (PF) domain. Among three subgroups, train drivers reported significantly lower scores on MCS and six health domains (excluding PF and bodily pain (BP)). After controlled confounders, conductors had significantly higher PCS and MCS scores than ground workers. There is heterogeneity on risk factors among three subgroups, but having long or irregular working schedules was negatively associated with HRQOL in all subgroups. SF-36v2 is a reliable and valid HRQOL measurement in railway workers in China. The frontline railway workers’ physical health was comparative to American norms, whilst mental health was relatively worse. Long or irregular working schedules were the most important factors.

## 1. Introduction

China’s total railway mileage reached 97,625 km by 2012, and is predicted to top 120,000 km by 2020, ranked second in the world after the United States [1]. As the world’s largest populated country and the fourth largest country by area, China has the highest rail transportation density in the world (39.95 million equated ton–km/km in 2012 [1]). The increasing traffic volume results in a heavy workload for about two million railway workers in China. Except for the heavy workload, railway workers, especially those who work on the front lines, have irregular work schedules. In addition, they have to work under poor working circumstances: high levels of noise, vibration, and being exposed to magnetic fields.

There is an increased concern about compromised health and wellbeing of railway workers. Studies conducted in developed countries have highlighted that irregular work hours could disrupt the sleep–wake cycle, and lead to sleepiness, fatigue, and performance impairment [2,3,4]. Occupational noise exposure could further contribute to sleep disturbance and poor mental health for railway workers, in addition to the hearing loss [5,6], whilst whole-body vibration exposure can cause low back pain and neck pain [7]. Furthermore, there is evidence supporting the association between magnetic fields and certain types of cancers in railway workers [8,9].

The majority of studies focusing on railway workers’ health in China have been limited to health examination results. It is found that the physical health of railway workers is not optimistic, with endocrinal and metabolic diseases and cardiovascular diseases (i.e., fatty liver, abnormal electrocardiogram, hypertension, hyperglycemia, and hyperlipidemia) being commonly detected [10,11]. Qiu et al. further pointed out that frontline railway workers’ general health conditions were even worse than others in the railway system [11]. Poor mental health of railway workers has also been reported; results from a meta-analysis found that railway workers’ mental health levels were lower than the national average, and it had not significantly changed during 1988–2009. Among different railway workers, locomotive drivers and conductors were more likely to suffer from mental disorders [12,13].

Health-related quality of life (HRQOL) is a self-perceived multi-dimensional construct that measures an individual’s health from physical, psychological, and social functioning [14]. Adopting HRQOL as a key outcome measure has gained increasing attention among occupational health professionals [15,16]. Currently, there are very limited empirical studies investigating HRQOL of railway workers internationally [17,18]. The Medical Outcomes Study 36-item Short Form (SF-36) is a well-known generic HRQOL instrument developed in the United States in the late 1980s. It has been evaluated and used in many populations around the world and most of the time its validity has been reported as satisfactory. However, in railway workers, the application of the SF-36 was seldom reported. This study will firstly validate the Short Form 36 version 2 (SF-36v2) scale, followed by investigating the HRQOL and associated factors of frontline railway workers in the Chinese population. Identifying HRQOL-associated factors will provide useful information for the implementation of relevant public health policies.

## 2. Materials and Methods

### 2.1. Subjects and Data Collection

Ankang is located in the central-northwest region of mainland China, and it is the most important transportation hub in the south of Shaanxi Province. The Ankang railway office is a subordinate unit of Xi’an Railway Bureau, which is responsible for the coordination among local agencies and railway units, and the safety supervision of local railway operations and production. In this study, Ankang Precinct refers to railway units in the Ankang area (including the Ankang railway locomotive depot, the Ankang train operation depot, the Ankang railway passenger transport section, etc.), which all relate to the Ankang railway office. In total, there are about 6000 railways in-service workers in the Ankang Precinct. Among 4000 workers working on the ground, nearly 60% (2400) are frontline workers, mainly responsible for the maintenance work and other related work at train stations. The remaining are mainly train conductors (about 1000) and drivers (about 1000), working on the train, and providing transport services. There were a total of 11 passenger trains sent out from Ankang station every day during the study period. According to the running time, there were four short-distance trains (one-way travel time less than 8 h), four middle-distance trains (one-way travel time around 12 h), and three long-distance trains (one-way travel time around 24 h). The short-distance trains need fewer workers (13–20 workers), and are mainly conductors, who are responsible for the management of passengers on the trains, whilst the long-distance trains need more workers (40–60 workers) in order to rotate.

**Figure 1:**
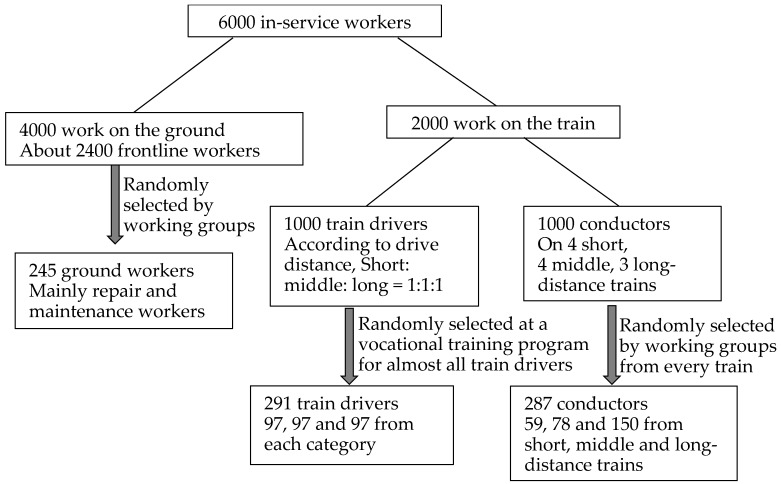
The sample selection process.

A stratified cluster sampling method was used. According to Halinski and Feldt [19], when using a multiple regression analysis, the ratio of observations to independent variables should be at least 10. Accordingly, the minimum sample size is estimated to be 150 for each subgroup (i.e., frontline ground workers, conductors, and train drivers). Considering the potential non-response or incomplete responses to the questionnaire, in this study we amplified the target sample size to be at least 200 for each subgroup. The detailed sample selection process is shown in Figure 1.

A cross-sectional survey was conducted in Ankang Precinct from January to April 2013. The Xi’an Jiaotong University Ethics Committee (No. 2012–189) granted permission to conduct this study. Written informed consent was obtained from each subject prior to the start of the study. A hard-copy structured questionnaire was self-completed by each respondent and then collected by researchers onsite. The completion of the questionnaire was checked immediately. When an incomplete questionnaire was found, respondents had the opportunity to complete missing items or opt to leave those questions unanswered. Fifty randomly selected frontline ground workers were invited to complete the identical questionnaire again one week after the initial survey.

### 2.2. Instruments

The questionnaire contains three main sections. First, the Chinese (mainland) SF-36v2 instrument [20]; Second, a series of socio-demographic information was included, including age, gender, educational level, marital status, having one or more children, personal monthly income, work type, and working schedule; and, finally, the last section covers health risk behaviors, including smoking and alcohol consumption history, and self-reported chronic disease status. A chronic disease checklist was included, which required respondents to indicate whether a doctor had ever told them that they had any of the following conditions: hypertension, diabetes, chronic lung disease (asthma, chronic bronchitis, and so on), heart disease, arthritis, mental disease (neurasthenia, clinical depression, or anxiety, and so on), kidney stone, chronic liver disease (hepatitis, fatty liver, and so on), and any other diagnosed chronic disease.

The SF-36v2 is one of the most widely used generic HRQOL measures in the world. It contains 36 items, except for the one single-item of health transition (HT) (item 2), the remaining 35 items could be grouped into eight health domains including physical functioning (PF), role-physical (RP), bodily pain (BP), general health (GH), vitality (VT), social functioning (SF), role-emotional (RE), and mental health (MH). The eight domains could be further aggregated into two summary measures: physical component summary (PCS) and mental component summary (MCS). The standard (four-week recall) Chinese (mainland) version, provided by Quality Metric Incorporated, was used in this study. The SF-36v2 was scored using Health Outcomes Scoring Software 4.5 (Quality Metric Incorporated, Lincoln, RI, USA). The 2009 USA general population norms were used as references due to the lack of general mainland Chinese population norms, but the equivalence of SF-36 scores in the Chinese population was proved later on in 2013, so we used this as a reference in this study [21]. Thus, all domains and summaries were calculated as norm-based scores which have the mean of 50 and standard deviation of 10. For all domains and summaries, higher scores indicate better HRQOL, whilst group-level scores less than 47 indicate being below the average range for the USA general population [20].

### 2.3. Statistical Analysis

The internal consistency reliability was measured with Cronbach’s α. The test–retest reliability, was assessed using the intraclass correlation coefficient (ICC). Generally, Cronbach’s α coefficients above 0.70 [22] and ICCs above 0.80 [23] indicate good internal and test–retest reliability, respectively. Validity analyses included convergent, discriminant, and construct validity. Convergent validity was examined using the correlation between items and their hypothesized domain. Discriminant validity was assessed by comparing the correlation of an item with its hypothesized domain and its correlation with other domains. A correlation greater than 0.40 was considered as a better convergent validity, whereas discriminant validity was supported when an item correlated significantly higher with its hypothesized health domain than with another domain [20]. Construct validity refers to the extent to which a test or survey, or a scale within a test or survey, measures a specific construct or trait, which was evaluated using confirmatory factor analysis (CFA). A hypothesized second-order factorial structure model was performed. Root mean square error of approximation (RMSEA) and goodness of fit index (GFI) were the two main absolute fit indices. The smaller the value of RMSEA is, the better the model fits. A RMSEA smaller than 0.05 suggested a good fit, 0.05–0.08 a fair fit, while greater than 0.10 suggested a poor fit. For GFI and some other relative fit indices, such as comparative fit index (CFI), normed fit index (NFI), the values close to 1 indicated a good model fit [24].

Railway workers were considered as a whole group, and were also divided into three subgroups: ground workers, conductors, and train drivers. Pearson’s chi-square test was used to compare differences in characteristics among the three subgroups. Scores were compared among the three subgroups and the same-gender subgroups using one-way analysis of variance (ANOVA) test. If significant differences were found, pairwise multiple comparisons were conducted to further examine differences between subgroups. Multivariable linear regression analyses were further performed to identify factors associated with HRQOL scores of railway workers. Independent variables included all of the characteristics shown in Table 1. Categorical variables were included in the form of dummy variables. All data analyses were carried out in SPSS 13.0 statistical software (SPSS Inc., Chicago, IL, USA), with *p*-value less than 0.05 as the statistically significant level.

## 3. Results

### 3.1. Characteristics of Respondents

Among 823 railway workers who initially agreed to participate, 39 workers dropped out of the study during the survey, leaving a final sample size of 784 respondents. The study sample consisted of 220 ground workers (mainly repair and maintenance workers), 281 conductors (57, 78, and 146 from short, middle, and long-distance trains, respectively), and 283 train drivers (including 93 short, 94 middle, and 96 long-distance drivers).

Table 1 shows the characteristics of the respondents. For the whole sample, 57.3% respondents were aged 30–44 years, 70.7% were male, and 65.0% have finished secondary school education. There were statistical differences in characteristics among three subgroups, except for marital status. Ground workers and conductors were older and had a lower monthly income than train drivers. All train drivers and 76.8% of ground workers were male, whilst conductors were mainly female (63.7%). As for the working schedule, ground workers worked mainly on day shifts, conductors mainly ran day and night shifts, whilst train drivers were mainly on night shifts.

### 3.2. Reliability and Validity of the SF-36v2

Internal consistency reliability and test–retest reliability were satisfactory. Cronbach’s α of the PCS and MCS components were 0.856 and 0.898, and 0.777–0.902 for the eight health domain scales. The ICCs of the PCS and MCS components were 0.913 and 0.804. For the eight domain scales, the ICCs were 0.744–0.912 (four domains fell between 0.744–0.795, while the other four were above 0.80). The item-scale correlation also showed satisfactory convergent and discriminant validity: all the correlations between items and their hypothesized domains were greater than 0.40, and 90.0% of items correlated significantly higher with its hypothesized health domain than with another domain. For the confirmatory factor analysis results, the RMSEA value was 0.083 and the GFI value was 0.769, while CFI, NFI and RFI values were all higher than 0.80, leading to acceptable construct validity.

### 3.3. HRQOL and Related Factors of Railway Workers

**Figure 2:**
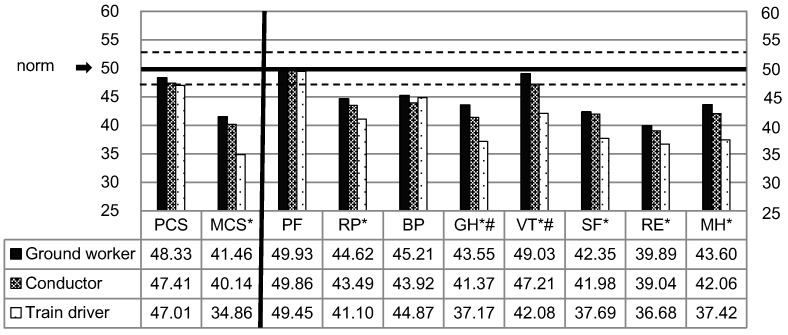
Mean norm-based scores of Short Form 36 version 2 (SF-36v2) health domains and component summary measures by group. Higher mean norm-based scores reflect better perceptions on the domains and summaries. The dashed lines indicate the upper (53) and lower (47) bounds of norm-based scores considered to be in the average range of functioning for group respondents; * statistical differences among three subgroups: ground workers and conductors both scored significantly higher than train drivers (*p* < 0.05); # statistical differences between the scores of conductors and ground workers (*p* < 0.05). BP: bodily pain; GH: general health; MCS: mental component summary; MH, mental health; PCS: physical component summary; PF: physical functioning; RE: role-emotional; RP: role-physical; SF: social functioning; VT, vitality.

Figure 2 presents the SF-36v2 profile of scores by subgroups. The PCS scores of three subgroups attained the average range for the USA general population, whereas the MCS scores were well below the range. On eight health domain scales, most domains scored below the norm. The only exception is the PF domain where all three subgroups scored around 50. Among the three subgroups, ground workers reported the highest scores on all summaries and domains, and conductors reported similar, but slightly lower scores than ground workers (significantly lower only on GH and VT domains). While the train drivers reported significantly lower scores on MCS and six health domains (excluding PF and BP, in which no significant difference was observed). As all of the train drivers, and 76.8%, of the ground workers were male, whilst conductors were mainly female, we made a further comparison among male subgroups and female subgroups. The results showed that among the three male subgroups, the train drivers still reported significantly lower scores on MCS and six health domains (excluding PF and BP) than male ground workers and conductors, while the female conductors reported significantly lower scores on both summaries and six health domains (excluding PF and RE) than female ground workers.

Multivariable linear regression analyses were further conducted to identify factors related to the HRQOL of frontline railway workers, and the results are shown in Table 2 (for physical HRQOL) and Table 3 (for mental HRQOL). After controlling for socio-demographic characteristics, health risk behaviors, and chronic disease status, conductors had significantly higher PCS and MCS scores than ground workers (*p* = 0.001), whilst the difference between train drivers and ground workers were positive, but insignificant. The most important factor that was associated with the HRQOL of frontline railway workers was working schedule. Having long or irregular working schedules (compared with mainly day shifts) was negatively associated with HRQOL in all subgroups, with magnitudes of significant negative coefficients consistently larger in mental health than physical health equations. Estimates on health status and health risk behaviors revealed that better health status (i.e., having no chronic disease) and lower health risk behavior (i.e., no alcohol consumption) were both associated with better HRQOL; however, the significant relationship between alcohol consumption and HRQOL was only limited to physical health. Other significant characteristics associated with physical health suggested that being younger (in the whole sample (but being older in ground workers)), having higher education (in ground workers and the whole sample), being richer (in ground workers, conductors, and the whole sample), being not married (in the train driver sample), and not having a child (in the ground worker sample) were significant and positively associated with physical health. Age was the only additional significant factor in the mental health equation in that being older was found to have a positive association on mental health in the ground worker sample. Gender and smoking status were found to be robustly insignificant.

## 4. Discussion

This study provides evidence for satisfactory reliability and validity of using the SF-36v2 to measure HRQOL of railway workers in China. As shown in the results, Cronbach’s α were all above 0.7 and ICCs were all around or above 0.8, indicating a good internal consistency reliability and test–retest reliability. Convergent, discriminant validity was satisfactory as well. The factor analysis, according to the recommended standards, showed acceptable construct validity.

Compared with the USA general population, it is evident that the frontline railway workers in Ankang Precinct suffered significant impairments to mental health. This result is contrary to a previous study conducted in Greece [18], which found no statistical difference on SF-36 scores between railway drivers and the Greek general population. However, this is consistent with the mental health survey results of railway workers in China, which reported poor mental health status compared with Chinese general population [12,13]. The SF-36v2 MCS measures about global mental health, with a low score on MCS, indicate frequent psychological distress, social, and role disability due to emotional problems [20]. The heavy workload, long or irregular work schedules, poor working circumstances, lack of job control, and social support from supervisors [25] can all act as chronic job stressors, which could lead to mental disorders, such as depression [26], social health impairments [27], or mental health complaints [28]. 

When comparing the HRQOL among three subgroups, train drivers reported significantly lower scores on MCS and six health domains. This may be due to the nature of their work as they often work alone in the locomotive cabin, and are busy operating the locomotive control system with no distraction allowed, in order to avoid signal passed at danger (SPAD) events or other related accidents [29]. In addition, their work hours are highly irregular, involve a high proportion of early mornings (starting before 06:00 a.m. and night shifts (ending after 04:00 a.m.). A previous study on train drivers showed that drivers experienced more stress, worse sleep quality, more sleepiness, lower job satisfaction, and also more social problems with their family than other comparable groups [30]. On the other hand, male ground workers and conductors reported similar scores, while female ground workers showed better HRQOL than female conductors. This may be due to shift work schedules doing more harm to women (female conductors in our study), who bear more responsibilities for housework and child care [31]. Interestingly, after controlling the confounding characteristics listed in Table 1, conductors had significantly better HRQOL than ground workers. Compared with the frontline ground workers, conductors’ work is relatively easy and within the sun-and-rain-free carriage, while the frontline repair and maintenance work requires more technique and is more energy consuming. Furthermore, the working environment is very harsh, as these workers have to walk along the railway lines on foot, saddled with heavy tools, no matter how hot or cold the weather is. Even so, whenever extreme weather comes, their workload will increase and will also be harder. When comparing the train driver and ground worker subgroups after controlling confounders, their respective HRQOL were almost the same, with insignificant positive coefficients. This may be due to the similar heavy workload and different, but equally difficult, working environments (train drivers work alone in a limited locomotive cabin, where they also deal with problems of eating, drinking, and excretion; on the other hand, frontline ground workers work outdoors facing tough conditions).

The most important factor associated with the HRQOL of frontline railway workers is their working schedule. Having long or irregular working schedules (compared with mainly day shifts) was negatively associated with HRQOL in all subgroups, with magnitudes of significantly negative coefficients consistently larger in mental health than physical health equations. Furthermore, it is the working schedule which causes the inconsistence of the HRQOL scores of the three subgroups before and after controlling confounders. This statement is substantiated by the fact that while we conducted the multivariable linear regression, we tried to put the confounders in the equation one by one, and every time we put the working schedule in, the inconsistence appeared. Long or irregular working schedules disrupt the sleep–wake cycle [2], leading to negative effects on sleep quality and performance. In addition, people who work irregular hours were more likely to report being emotionally exhausted [32]. A study involving night-shift, day-shift, and non-shift workers showed that night-shift workers reported a greater percentage of unhappy time. In analyses of quality of life, night-shift workers were less satisfied with domains of spiritual ‘being’ and physical and community ‘belonging’. They also reported having fewer opportunities to improve their physical ‘being’, leisure, and personal growth than the other two groups [33]. Though it is not possible to avoid night shifts in 24-h rail transportation, it is possible to adjust shift lengths or reduce the number of consecutive nights of shiftwork, and to improve facilities to reduce the harm done by the shift system.

Other significant personal characteristics associated with HRQOL include age, education level, marital status, having one or more children, personal income, alcohol consumption, and chronic disease status. The signs of significant characteristics are mostly in line with the literature. Chronic diseases can influence an individual’s HRQOL directly, while alcohol consumption can increase an individual’s risk of serious health problems and impact on HRQOL in an indirect, harmful way through chronic diseases [34]. Having a higher educational level means more knowledge to live healthy life, and more social and psychological resources to become healthy [35]. Higher income favors health through the material conditions necessary for biological survival, and through social participation and opportunity to control life circumstances [36]. Occupational health care providers should make further efforts on education and health promotion, to strengthen workers’ health consciousness and chronic disease prevention capacity.

The association between age and HRQOL was found to be significant only in some age groups among ground workers. Differing from the other two groups, there exists a positive association between age and HRQOL. The potential reason may be that as ground workers grow older, they became more conscious of practicing healthy behaviors (i.e., routine bedtime, regular hours of sleep, regularity of meals and physical activity) and this leads to the better HRQOL. However, for conductors and train drivers, due to the nature of their shift-work, they had less opportunity to engage in healthy behaviors. In addition, aging could decrease their shift-work tolerance [37]. Having a child was significant and negatively associated with physical HRQOL in ground workers and this may be owing to their responsibility of taking care of children after work. For conductors and train drivers, both need to work on the train and stay away from home, and, therefore, the key responsibility of child raising may lay with their partners. It is also worth noting that, for train drivers, being married was associated with poor HRQOL, although the relationship was only significant in the physical HRQOL domain. So far the relationship between marital status and HRQOL is inconclusive with the majority of studies finding being married is associated with a better HRQOL [38]. However, a study on Chinese railway workers also reported that being married was negatively associated with self-rated health [17]. Considering train workers’ heavy workload and long continuous working hours, the family-work conflict could be severe in this group, and the conflict could further reduce their physical health [39]. These results highlight that some family-friendly policies, like a flexible schedule and parental/family leave, or on-site or near-site childcare centers are needed to improve work–life balance.

Although our study was done in only one city, due to the use of a stratified cluster sampling method, it depicts the attributes of frontline railway workers in the province and other larger units of the country. It can, therefore, reflect the HRQOL of frontline railway workers in China to a certain degree, as all the railway bureaus in China are under the centralized management of the Chinese Ministry of Railways, and all of these units work similarly in terms of their administrative and technical bases. Moreover, our study results show that long or irregular working schedules impair workers’ HRQOL, which is in line with the findings of previous studies. However, the inconsistency of the HRQOL scores of the three subgroups before and after controlling confounders may be a new finding of our study, which indicated that workload or working environment may be the potential cause of compromised HRQOL in frontline railway workers. A previous study, analyzing the attitudes and opinions of railway signalers and related staff, found that those workers doing less complex work had more positive perceptions, more job satisfaction, and less stress [40]. Thus, further research should take these factors into consideration of compromised HRQOL in frontline railway workers.

There were two major limitations in our study. First, it would be ideal to compare the SF-36 results with the norm of the Chinese general population; however, this was unavailable. Second, as this is a cross-sectional study, all relationships reported in the paper should be explained as association rather than causality.

## 5. Conclusions

SF-36v2 is a satisfactory, reliable and valid HRQOL measurement tool for railway workers in China. We described that frontline railway workers’ physical health was similar to that of the USA general population, whilst the mental health was relatively worse. Among the three subgroups there was an inconsistency of the HRQOL scores before and after controlling confounders. There is heterogeneity of risk factors among three subgroups, but long or irregular working schedules were the most important factors.

## Figures and Tables

**Table 1:** Characteristics of participants ^#^.

	Overall (*N* = 784)	Ground Worker (*n* = 220)	Conductor (*n* = 281)	Train Driver (*n* = 283)
Age (years, %) *				
18–29	19.0	22.6	11.2	23.9
30–44	57.3	41.0	72.8	54.3
45–60	23.7	36.3	15.9	21.7
Gender (%) *				
Male	70.7	76.8	36.3	100.0
Female	29.3	23.2	63.7	0.0
Educational level (%) *				
Compulsory	8.4	17.0	7.7	2.5
Secondary	65.0	52.3	77.4	62.8
Tertiary	26.6	30.7	15.0	34.8
Marital status (%)				
In marriage	79.8	79.4	83.2	76.9
Others	20.2	20.6	16.8	23.1
Having one or more children (%) *				
Yes	79.6	78.8	88.6	71.6
No	20.4	21.2	11.4	28.4
Personal monthly income (CNY, %) *				
<2500	32.5	22.4	60.4	11.2
2500–3499	33.0	58.0	32.2	14.0
≥3500	34.5	19.5	7.4	74.8
Working schedule (%) *				
Mainly day shifts	27.0	79.7	7.6	5.0
Day and night shifts	30.0	18.4	60.4	9.0
Mainly night shifts	36.6	1.8	26.9	73.1
Usually work more than 24 h	6.5	0.0	5.1	12.9
Smoking (%) *				
No	59.7	55.0	78.2	44.9
Yes	40.3	45.0	21.8	55.1
Alcohol consumption (%) *				
No	62.3	61.6	75.3	49.8
Yes	37.7	38.4	24.7	50.2
Chronic disease (%) *				
No	64.9	61.2	60.1	72.4
Yes	35.1	38.8	39.9	27.6

^#^ Eleven missing on age, seven missing on marital status, nine missing on whether having one or more children, and eighteen missing on personal monthly income; * statistical difference among three subgroups based on χ^2^ test (*p* < 0.05).

**Table 2:** Multivariable linear regression on physical component summary scores of SF-36v2 ^#^.

Independent Variables	Overall		Ground Worker		Conductor		Train Driver	
	Coefficient	*p*-Value	Coefficient	*p*-Value	Coefficient	*p*-Value	Coefficient	*p*-Value
Work type								
Ground worker	Ref.	–						
Conductor	3.198	0.001						
Train driver	1.958	0.076						
Age (years)								
18–29	Ref.	–	Ref.	–	Ref.	–	Ref.	–
30–44	−1.090	0.325	3.948	0.045	−2.641	0.223	−1.644	0.435
45–60	−2.676	0.031	1.367	0.519	−4.620	0.054	−2.601	0.278
Gender								
Male	Ref.	–	Ref.	–	Ref.	–	Ref.	–
Female	−0.361	0.640	0.754	0.570	−0.638	0.596	–	–
Educational level								
Compulsory	Ref.	–	Ref.	–	Ref.	–	Ref.	–
Secondary	0.821	0.384	2.036	0.139	−0.179	0.906	0.029	0.992
Tertiary	3.549	0.001	3.553	0.028	2.439	0.173	2.457	0.445
Marital status								
In marriage	Ref.	–	Ref.	–	Ref.	–	Ref.	–
Others	1.162	0.212	−1.341	0.433	−0.328	0.813	4.485	0.037
Having one or more children								
Yes	Ref.	–	Ref.	–	Ref.	–	Ref.	–
No	0.559	0.623	5.303	0.018	−0.044	0.985	−1.412	0.415
Personal monthly income (CNY)								
<2500	Ref.	–	Ref.	–	Ref.	–	Ref.	–
2500–3500	0.862	0.202	1.084	0.405	0.668	0.462	2.177	0.222
≥3500	1.821	0.030	4.098	0.014	3.629	0.022	1.447	0.414
Working schedule								
Mainly day shifts	Ref.	–	Ref.	–	Ref.	–	Ref.	–
Day and night shifts	−3.092	0.001	−3.492	0.011	−2.204	0.198	−2.290	0.323
Mainly night shifts	−5.696	<0.001	0.707	0.858	−4.105	0.021	−6.704	0.001
Usually work more than 24 h	−7.402	<0.001	–	–	−7.092	0.006	−7.561	0.001
Smoking								
No	Ref.	–	Ref.	–	Ref.	–	Ref.	–
Yes	0.556	0.358	−0.657	0.563	0.446	0.725	1.017	0.267
Alcohol consumption								
No	Ref.	–	Ref.	–	Ref.	–	Ref.	–
Yes	−1.205	0.038	−0.875	0.425	−0.784	0.484	−1.480	0.100
Chronic disease								
No	Ref.	–	Ref.	–	Ref.	–	Ref.	–
Yes	−3.271	<0.001	−4.628	<0.001	−3.447	<0.001	−2.075	0.044

^#^ Train drivers were all male; no ground workers usually worked more than 24 h; Ref.: reference group.

**Table 3:** Multivariable linear regression on mental component summary scores of SF-36v2 ^#^.

Independent Variables	Overall		Ground Worker		Conductor		Train Driver	
	Coefficient	*p*-Value	Coefficient	*p*-Value	Coefficient	*p*-Value	Coefficient	*p*-Value
Work type								
Ground worker	Ref.	–						
Conductor	5.291	0.001						
Train driver	2.126	0.242						
Age (years)								
18–29	Ref.	–	Ref.	–	Ref.	–	Ref.	–
30–44	−2.013	0.270	5.620	0.066	−1.206	0.715	−6.905	0.063
45–60	−0.392	0.848	7.045	0.034	−1.568	0.666	−4.156	0.324
Gender								
Male	Ref.	–	Ref.	–	Ref.	–	Ref.	–
Female	2.089	0.100	3.127	0.132	2.052	0.264	–	–
Educational level								
Compulsory	Ref.	–	Ref.	–	Ref.	–	Ref.	–
Secondary	−0.589	0.705	0.466	0.827	−1.333	0.564	0.349	0.948
Tertiary	1.472	0.400	3.901	0.121	−4.140	0.130	3.864	0.495
Marital status								
In marriage	Ref.	–	Ref.	–	Ref.	–	Ref.	–
Others	−0.825	0.590	−2.080	0.435	−3.675	0.084	1.071	0.776
Having one or more children								
Yes	Ref.	–	Ref.	–	Ref.	–	Ref.	–
No	3.124	0.095	5.427	0.118	4.705	0.180	1.564	0.608
Personal monthly income (CNY)								
<2500	Ref.	–	Ref.	–	Ref.	–	Ref.	–
2500–3500	−1.057	0.342	1.285	0.526	−1.267	0.360	−2.774	0.376
≥3500	1.198	0.386	3.624	0.158	3.942	0.101	1.296	0.678
Working schedule								
Mainly day shifts	Ref.	–	Ref.	–	Ref.	–	Ref.	–
Day and night shifts	−5.651	<0.001	−4.490	0.034	−5.332	0.042	−4.718	0.247
Mainly night shifts	−11.569	<0.001	−14.116	0.023	−9.370	0.001	−12.486	<0.001
Usually work more than 24 h	−12.527	<0.001	–	–	−14.898	<0.001	−12.480	0.002
Smoking								
No	Ref.	–	Ref.	–	Ref.	–	Ref.	–
Yes	0.979	0.326	0.523	0.767	−0.906	0.640	2.856	0.077
Alcohol consumption								
No	Ref.	–	Ref.	–	Ref.	–	Ref.	–
Yes	−1.250	0.190	0.242	0.887	1.324	0.438	−2.483	0.116
Chronic disease								
No	Ref.	–	Ref.	–	Ref.	–	Ref.	–
Yes	−2.068	0.023	−6.448	<0.001	−2.828	0.030	1.535	0.396

^#^ Train drivers were all male; no ground worker usually worked more than 24 h.

## References

[1] Qian M. (2013). China Railway Yearbook.

[2] Tepas D.I., Carvalhais A.B. (1989). Sleep patterns of shiftworkers. Occup. Med..

[3] Harma M. (2002). The effect of an irregular shift system on sleepiness at work in train drivers and railway traffic controllers. J. Sleep Res..

[4] Dorrian J., Baulk S.D., Dawson D. (2011). Work hours, workload, sleep and fatigue in Australian Rail Industry employees. Appl. Ergon..

[5] enHealth Council (2004). The Health Effects of Environmental Noise—Other Than Hearing Loss.

[6] Landon P., Breysse P., Chen Y. (2005). Noise exposures of rail workers at a North American chemical facility. Am. J. Ind. Med..

[7] McBride D., Paulin S., Herbison G.P., Waite D., Bagheri N. (2014). Low back and neck pain in locomotive engineers exposed to whole-body vibration. Arch. Environ. Occup. Health.

[8] Alfredsson L., Hammar N., Karlehagen S. (1996). Cancer incidence among male railway engine-drivers and conductors in Sweden, 1976–1990. Cancer Causes Control.

[9] Floderus B., Trnqvist S., Stenlund C. (1994). Incidence of selected cancers in Swedish railway workers, 1961–1979. Cancer Causes Control.

[10] Huang X. (2013). Health status analysis of 15,986 railway workers. Chin. Community Dr..

[11] Qiu X., Yan H., Hu Y. (2007). Health status of railway staff and workers:Analysis of health examinations. Chin. J. Health Educ..

[12] Yi X., Liu Y., Liao J., Dou D., Peng K. (2010). Changes in Chinese Railwayman’s Mental Health (1988–2009): A Cross-Temporal Meta-Analysis. J. Beijing Jiaotong Univ. (Social. Sci. Ed.).

[13] Lv B., Tang S., Zhong S., Pan J. (2011). Meta-analysis on mental health status of railway locomotive conductors. Chin. J. Ind. Med..

[14] Guyatt G., Feeny D., Patrick D. (1991). Issues in quality-of-life measurement in clinical trials. Control. Clin. Trials.

[15] Blanc P.D. (2004). Why quality of life should matter to occupational health researchers. Occup. Environ. Med..

[16] Chattopadhyay K., Chattopadhyay C., Kaltenthaler E. (2014). Health-related quality-of-life of coal-based sponge iron plant workers in Barjora, India: A cross-sectional study. BMJ Open.

[17] Cui J., Wang Y., Qin L., Huo W., He Z., Hu Y., Xue S., Lv S. (2011). Self-rated Health Status and Related Factors of Workers in Key Sectors of Railway System. J. Environ. Occup. Med..

[18] Nena E., Tsara V., Steiropoulos P., Constantinidis T., Katsarou Z., Christaki P., Bouros D. (2008). Sleep-disordered breathing and quality of life of railway drivers in Greece. Chest.

[19] Halinski R.S., Feldt L.S. (1970). The selection of variables in multiple regression analysis. J. Educ. Meas..

[20] Maruish M.E. (2011). User’s Manual for the SF-36v2 Health Survey.

[21] Maudrene L.S.T., Hwee-Lin W., Jeannette L., Stefan M., Derrick H., E-Shyong T., Julian T. (2013). The Short Form 36 English and Chinese versions were equivalent in a multiethnic Asian population. J. Clin. Epidemiol..

[22] Nunnally J.C., Bernstein I. (1994). Psychometric Theory.

[23] Landis J.R., Koch G.G. (1977). The measurement of observer agreement for categorical data. Biometrics.

[24] Hu L., Bentler P.M. (1999). Cutoff criteria for fit indexes in covariance structure analysis: Conventional criteria versus new alternatives. Struct. Eq. Model. A Multidiscip. J..

[25] Piros S., Karlehagen S., Lappas G., Wilhelmsen L. (2000). Psychosocial risk factors for myocardial infarction among Swedish railway engine drivers. J. Cardiovasc. Risk.

[26] Evans D., Mallet L., Flahault A., Cothereau C., Velazquez S., Capron L., Lejoyeux M. (2013). The importance of both workplace and private life factors in psychological distress: A large cross-sectional survey of French railway company employees. Soc. Psychiatry Psychiatr. Epidemiol..

[27] Ku C., Smith M.J. (2010). Organisational factors and scheduling in locomotive engineers and conductors: Effects on fatigue, health and social well-being. Appl. Ergon..

[28] Zoer I., Ruitenburg M.M., Botje D., Frings-Dresen M.H.W., Sluiter J.K. (2011). The associations between psychosocial workload and mental health complaints in different age groups. Ergonomics.

[29] Naweed A. (2013). Psychological factors for driver distraction and inattention in the Australian and New Zealand rail industry. Accid. Anal. Prev..

[30] Kecklund L., Ingre M., Kecklund G., Söderström M. The TRAIN-Project: Railway safety and the train driver information environment and work situation—A summary of the main results. Proceedings of the Signalling Safety 2001.

[31] Siraj R., Ravichandran N. (2012). Shift Work: Evaluation of Employee’s well being and its impact on Quality of Life. Int. J. Exclus. Manag. Res..

[32] Zwieten M. (2012). Impact of Irregular Working Hours.

[33] Lipovcan L.J.K., Larsen Z.P., Zganec N. (2004). Quality of life, life satisfaction and happiness in shift- and non-shiftworkers. Rev. Saúde Pública.

[34] Rehm J., Room R., Graham K., Monteiro M., Gmel G., Sempos C.T. (2003). The relationship of average volume of alcohol consumption and patterns of drinking to burden of disease: An overview. Addiction.

[35] Ross C.E., Wu C. (1995). The Links Between Education and Health. Am. Sociol. Rev..

[36] Marmot M. (2002). The influence of income on health: Views of an epidemiologist. Health Aff..

[37] Harma M. (1996). Ageing, physical fitness and shiftwork tolerance. Appl. Ergon..

[38] Taghavi S.M., Mokarami H., Nazifi M., Choobineh A. (2014). The Influence of Socio-Demographic, Health and Work-Related Factors on Health-Related Quality of Life among Iranian Industrial Workers. Health (Irvine Calif.).

[39] Frone M.R., Russell M., Cooper M.L. (1997). Relation of work-family conflict to health outcomes: A four-year longitudinal study of employed parents. J. Occup. Organ. Psychol..

[40] Ryan B., Wilson J.R., Sharples S., Clarke T. (2009). Attitudes and opinions of railway signallers and related staff, using the Rail Ergonomics Questionnaire (REQUEST). Appl. Ergon..

